# Crystal structure and Hirshfeld surface analysis of dimethyl 3,3′-{[(1*E*,2*E*)-ethane-1,2-diyl­idene]bis(aza­nylyl­idene)}bis­(4-methyl­benzoate)

**DOI:** 10.1107/S2056989022002092

**Published:** 2022-03-01

**Authors:** Semanur Yeşilbağ, Emine Berrin Çınar, Necmi Dege, Erbil Ağar, Eiad Saif

**Affiliations:** a Department of Chemistry, Faculty of Arts and Sciences, Ondokuz Mayıs University, Samsun, 55200, Turkey; b Department of Physics, Faculty of Arts and Sciences, Ondokuz Mayıs University, Samsun, 55200, Turkey; c Department of Computer and Electronic Engineering Technology, Sanaa Community, College, Sanaa, Yemen; d Department of Electrical and Electronic Engineering, Faculty of Engineering, Ondokuz Mayıs University, 55139, Samsun, Turkey

**Keywords:** aza­nylyl­idene, crystal structure, electrostatic potential map, energy frameworks, Hirshfeld, methyl­benzoate, shape index, curvedness

## Abstract

The title Schiff base compound, C_20_H_20_N_2_O_4_, synthesized by the condensation reaction of methyl 3-amino-4-methyl­benzoat and glyoxal in ethanol, crystallizes in the the monoclinic space group *P*2_1_/*n*. The mol­ecule is Z-shaped with the C—N—C—C torsion angle being 47.58 (18)°. In the crystal, pairs of mol­ecules are linked *via* C—H⋯N hydrogen bonds, forming centrosymetric dimers with an *R*_2_^2^(8) ring motif; this connectivity leads to the formation of columns running along the *a-*axis direction. Hirshfeld surface analysis and two-dimensional fingerprint plots were used to explore the inter­molecular inter­actions and revealed that the most significant contributions to the crystal packing are from H⋯H (49.4%), H⋯O/O⋯H (19.0%) and H⋯C/C⋯H (17.5%) contacts. Energy frameworks were constructed through different inter­molecular inter­action energies to investigate the stability of the compound. The net inter­action energies for the title compound were found to be electrostatic (*E*_ele_ = −48.4 kJ mol^−1^), polarization (*E*_pol_ = −9.7 kJ mol^−1^), dispersion (*E*_dis_ = −186.9 kJ mol^−1^) and repulsion (*E*_rep_ = 94.9 kJ mol^−1^) with a total inter­action energy, *E*_tot_, of −162.4 kJ mol^−1^.

## Abstract 2

The title Schiff base compound, synthesized by the condensation reaction of methyl 3-amino-4-methyl­benzoat and glyoxal in ethanol, crystallizes in the the monoclinic space group *P*2_1_/*n*. The mol­ecule is Z-shaped with the C—N—C—C torsion angle being 47.58 (18)°. In the crystal, pairs of mol­ecules are linked *via* C—H⋯N hydrogen bonds, forming centrosymetric dimers with an 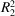 (8) ring motif; this conectivity leads to the formation of columns running along the *a-*axis direction.

## Chemical context

In this study, the title Schiff base compound was synthesized by the condensation reaction of methyl 3-amino-4-methyl­benzoat and glyoxal in ethanol. Schiff bases are studied widely because of their synthetic flexibility, selectivity and sensitivity towards the central metal atom, structural similarities with natural biological compounds and because of the presence of an azomethine group (–N=CH–), which is important for elucidating the mechanism of the transformation and racem­ization reaction biologically (Sharghi *et al.*, 2003 ▸). Schiff bases having chelation with oxygen and nitro­gen donors and their complexes have been used as drugs and are reported to possess a wide variety of biological activities against bacteria, fungi and certain types of tumors; in addition, they have many biochemical, clinical and pharmacological properties (Przybylski *et al.*, 2009 ▸; Barbosa *et al.*, 2020 ▸). In recent years, these mol­ecules, which belong to a large family of click reactions, have attracted a lot of inter­est for their role in the development of self-healing hydro­gels (Xu *et al.*, 2019 ▸) . Over the past few years, some metal complexes of Schiff bases have attracted great inter­est in many fields. The binding inter­actions of metal complexes with DNA have been studied (Shahabadi *et al.*, 2010 ▸). Schiff bases have different applications in many research areas including organic, inorganic, biological and materials chemistry (Fan *et al.*, 2020 ▸) and as dyes for the textile and related industries. These compounds also have unique characteristics that make them promising candidates for photovoltaic and photonic materials applications (Abdel-Shakour *et al.*, 2019 ▸; Imer *et al.*,2018 ▸). We report herein XRD data and Hirshfeld surface analysis of a new Schiff base compound, dimethyl 3,3′-{[(1*E*,2*E*)-ethane-1,2-diyl­idene]bis­(aza­nylyl­idene)}bis­(4-methyl­benzoate), for which energy frameworks of the crystal packing were calculated. 
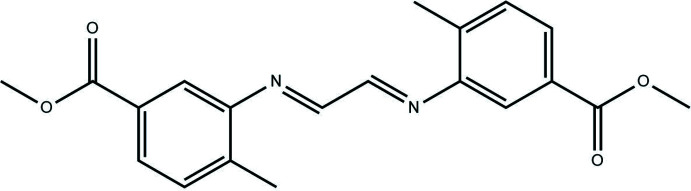


## Structural commentary

**Figure 1:**
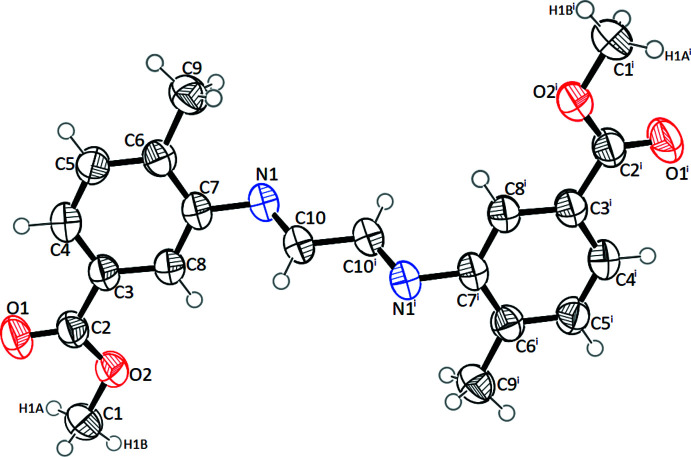
The mol­ecular structure of the title compound, showing the atom labelling. Displacement ellipsoids are drawn at the 40% probability level.

The mol­ecular structure of the title complex is illustrated in Fig. 1 ▸. The mol­ecule is located in a special position related to the inversion centre 8*i* (*mm*2) at the middle of the C10—C10^i^ bond [symmetry code: (i) 1 − *x*, 1 − *y*, 1 − *z*]. The mol­ecule is Z-shaped with the C10—N1—C7—C8 torsion angle being 47.58 (18)°. The benzene rings are located in planes parallel to each other. The values of the C1—O2, O2—C2 and C2—O1 bond lengths and the O1—C2—O2, C2—O2—C1 bond angles are close to those reported for similar complexes (see *Database survey*). Some selected geometric parameters of the mol­ecule are given in Table 1 ▸. The azomethine C=N bond length is 1.2713 (17) Å, which is quite close to the corresponding values reported by Gumus *et al.* (2021 ▸) and Kansiz *et al.* (2021 ▸) [1.276 (6) and 1.287 (6) Å and 1.287 (5) Å, respectively].

## Supra­molecular features

**Figure 2:**
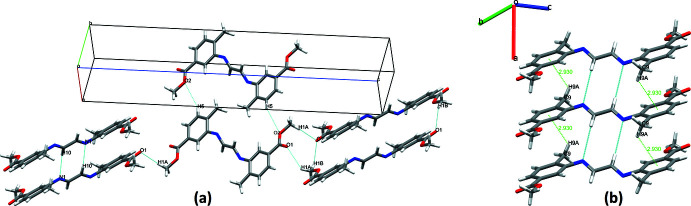
A view of the crystal packing of the title compound.

Although no classical hydrogen bonds are found in the crystal structure, weak hydrogen bonds are present (Table 2 ▸, Fig. 2 ▸). The role of hydrogen bonds in the formation of the crystal lattice is shown in Fig. 2 ▸ *a*. Pairs of mol­ecules form inversion dimers with an R_2_^2^(8) ring motif *via* C10—H10⋯N1 hydrogen bonds, leading to the formation of columns running along the *a*-axis direction. A weak C9—H9*A*⋯*Cg*1 contact is also present (Table 2 ▸), which reinforces the crystal structure and plays a major role in the supra­molecular framework stabilization, see Fig. 2 ▸ *b*.

## Database survey

A search of the Cambridge Structural Database (CSD, version 5.40, update of August 2020; Groom *et al.*, 2016 ▸) found a structure that is very similar to the title compound, *viz.*2-(4′-carbometh­oxy-2′-nitro­benz­yl)-1,3,5-tri­methyl­benzene (CBYMBZ; van der Heijden *et al.*, 1975 ▸). In CBYMBZ, the bond lengths and bond angles for the methyl formate are: C8—O4 = 1.448 (4) Å, O4—C7 = 1.326 (3) Å, C7—O3 = 1.193 (3) Å, C8—O4—C7 = 116.2 (3)° and O4—C7—O3 = 123.9 (2)°.

## Hirshfeld surface analysis

**Figure 3:**
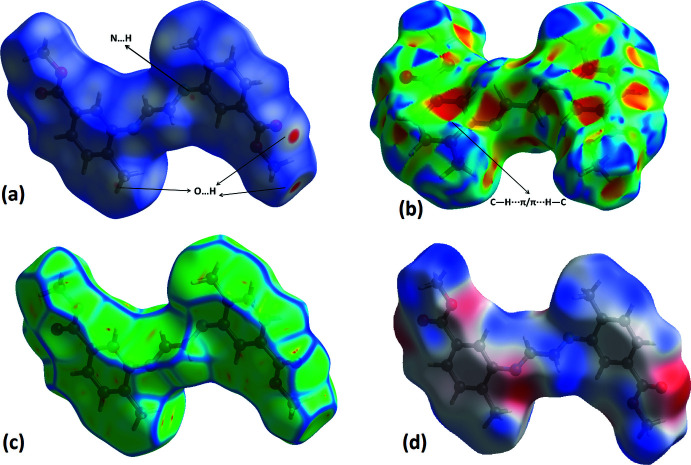
The Hirshfeld surface of the title compound mapped over (*a*) *d*_norm_, (*b*) shape-index, (*c*) curvedness and (*d*) electrostatic potential.

The inter­molecular inter­actions present in the crystal structure were visualized by drawing contact and shape descriptors using *Crystal Explorer17.5* (Turner *et al.*, 2017 ▸). The Hirshfeld surfaces mapped over *d*_norm_, curvedness, shape-index and electrostatic potential are shown in Fig. 3 ▸. The mol­ecular Hirshfeld surfaces were calculated using a standard (high) surface resolution and with the three-dimensional *d*_norm_ surfaces mapped over a fixed colour scale from −0.083 (red) to 1.171 (blue) a.u. Red spots in Fig. 3 ▸ *a* correspond to the near-type H⋯O contacts resulting from C—H⋯O and N—H⋯O hydrogen bonds. The shape-index surface (Fig. 3 ▸ *b*) shows red concave regions with ‘bow-tie’ patterns, indicating the presence of aromatic stacking inter­actions (C—H⋯π). In Fig. 3 ▸ *c*, the curvedness plots show flat surface patches characteristic of planar stacking. The mol­ecular properties can be described by mapping the mol­ecular electrostatic potential (−0.067 to 0.025 a.u.), which plays a key role in identifying reactive positions on the mol­ecular surface. The Fig. 3 ▸ *d* map is useful for predicting the position of nucleophile and electrophile attacks. The blue and red regions observed on the surface around the different atoms correspond to positive and negative electrostatic potentials, respectively. It shows clearly that the electron-rich sites are mainly localized around the oxygen atoms.

**Figure 4:**
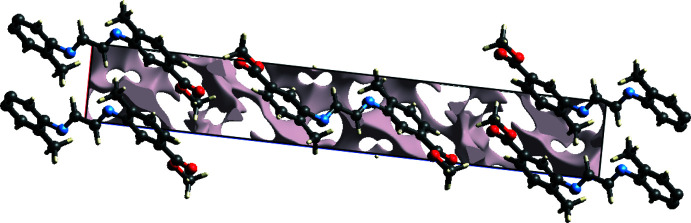
A view of the crystal voids.

**Figure 5:**
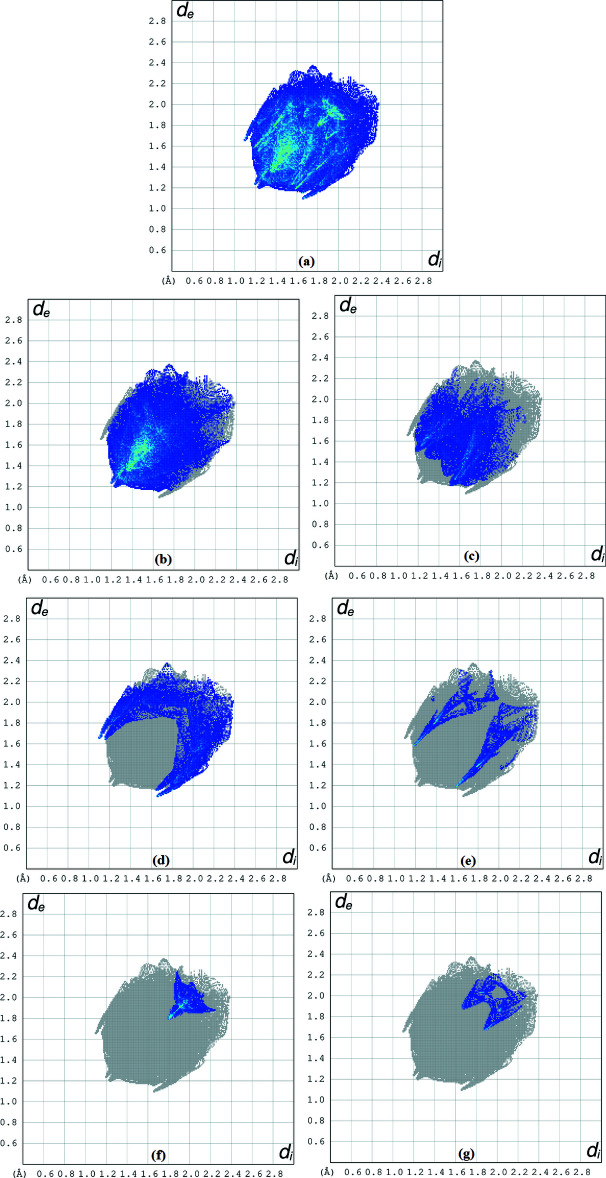
The two-dimensional fingerprint plots for (*a*) all inter­actions and those delineated into (*b*) H⋯H, (*c*) H⋯O/O⋯H, (*d*) H⋯C/C⋯H, (*e*) H⋯N/N⋯H, (*f*) C⋯C and (*g*) C⋯O/O⋯C contacts.

Inter­molecular contacts and the location of electron-rich regions provide an indication of the stacking in the crystal. To understand this stacking, the crystal voids [calculated with *Crystal Explorer17.5* (Turner *et al.*, 2017 ▸)] were visualized (Fig. 4 ▸). The void parameters of the title compound give a void volume of 76.77 Å^3^, an area of 340.15 Å^2^, a globularity of 0.257 and asphericity value of 0.807. Fig. 5 ▸ *a* shows the two-dimensional fingerprint plot of the sum of all the contacts contributing to the Hirshfeld surface represented in normal mode. The H⋯H contacts make the largest contribution to the overall crystal packing at 49.4%. This contribution arises as widely scattered points of high density due to the large hydrogen content of the mol­ecule with the two tips at *d*_e_ + *d*_i_ = 2.43 Å (Fig. 5 ▸ *b*). Scattered points of the H⋯O/O⋯H inter­actions contribution (19.0%) have a tip at *d*_e_ + *d*_i_ = 2.68 Å. (Fig. 5 ▸ *c*) . The pair of characteristic wings in Fig. 5 ▸ *d* arise from H⋯C/C⋯H contacts (17.5%) and pairs of spikes are observed with the tips at *d*_e_ + *d*_i_ = 2.75 Å and 2.80 Å. The H⋯N/N⋯H contacts, contributing 6.3% to the Hirshfeld surface, are also represented by a pair of sharp spikes at *d*_e_ + *d*_i_ = 2.76 Å, Fig. 5 ▸ *e*. As seen in Fig. 5 ▸ *f*, the C⋯C contacts (4.9%) have an arrow-shaped distribution of points with its tip at *d*_e_ = *d*_i_ = 3.59 Å. The contribution of the C⋯O/O⋯C contacts to the Hirshfeld surface (2.9%) is negligible, Fig. 5 ▸ *g*.

## Inter­action energies

**Figure 6:**
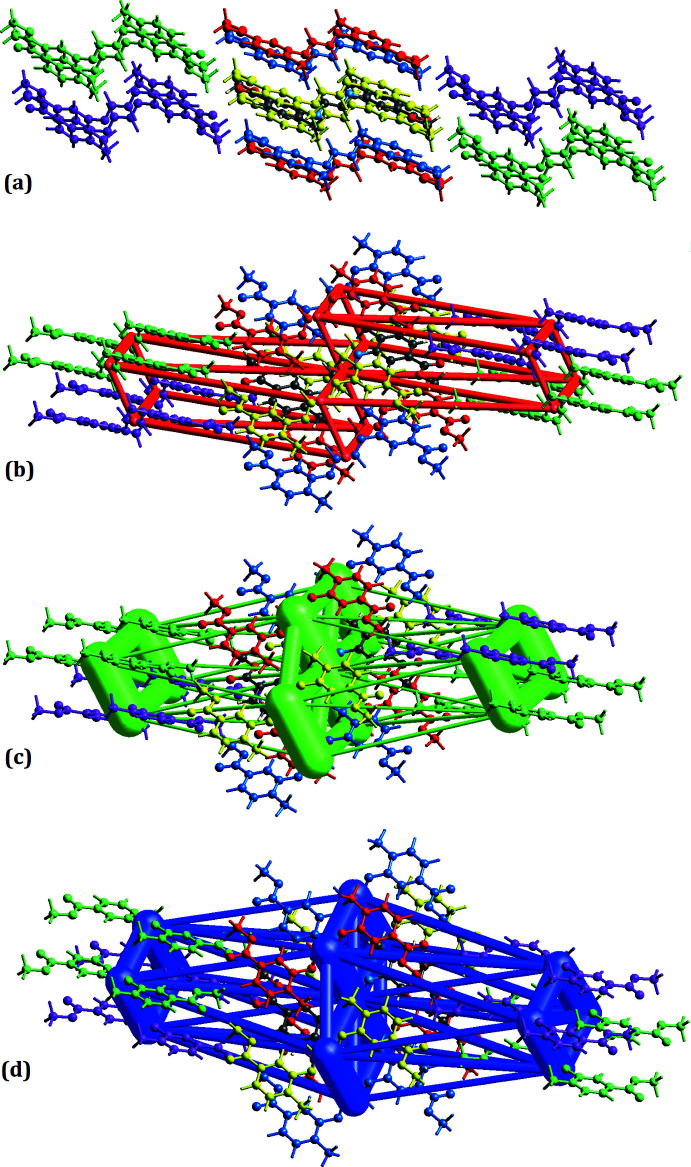
Inter­molecular inter­action energies: (*a*) Color coding of neighboring molecules with respect to the central molecule (gray), (*b*) Coulombic, (*c*) dispersion and (*d*) total inter­action energy for the title compound.

Inter­action energies for the title compound were calculated using the CE-B3LYP/6-31G(d,p) quantum level of theory, as available in *CrystalExplorer* (Turner *et al.*, 2017 ▸). The total inter­molecular inter­action energy (*E*_tot_) is the sum of four energy terms: electrostatic (*E*_ele_), polarization (*E*_pol_), dispersion (*E*_disp_) and exchange-repulsion (*E*_rep_) with scale factors of 1.057, 0.740, 0.871 and 0.618, respectively. The relative strengths of the inter­action energies in individual directions are represented by cylinder-shaped energy frameworks. The energy-framework calculations were analysed to understand the topologies of the pair-wise inter­molecular inter­action energies. The energy framework is constructed to compare the different energy components, *i.e.* repulsion (*E*_rep_), electrostatic (*E*_ele_), dispersion (*E*_dis_), polarization (*E*_pol_) and total (*E*_tot_) energy (Mackenzie *et al.*, 2017 ▸). The energies between mol­ecular pairs are indicated as cylinders joining the centroids of pairs of mol­ecules with the thickness of the cylinder radius being directly proportional to the amount of inter­action energy between the pair of mol­ecules (Wu *et al.*, 2020 ▸). As seen in Fig. 6 ▸, the red mol­ecule with symmetry (*x*, *y*, *z*) located at a distance of 4.60 Å from the centroid of the selected mol­ecule has shown the highest total inter­action energy of −63.7 kJ mol^−1^, whereas the purple mol­ecule at the symmetry position (−*x* + 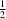 , *y* + 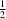 , −*z* + 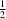 ) located at a distance of 15.88 Å from the centroid of the selected mol­ecule has the lowest total inter­action energy of −13.4 kJ mol^−1^. The net inter­action energies for the title compound are electrostatic (*E*_ele_) = −48.4 kJ mol^−1^, polarization (*E*_pol_) = −9.7 kJ mol^−1^, dispersion (*E*_dis_) = −186.9 kJ mol^−1^, repulsion (*E*_rep_) = 94.9 kJ mol^-1 and^ total inter­action energy (*E*_tot_) = −162.4 kJ mol^−1^. The dispersion energy is dominant.

## Synthesis and crystallization

27.3 mg (0.165 mmol) of 2-amino-3-methyl­phenol were dissolved in 20 ml of ethanol. To this was added 11.98 mg (0.083 mmol) of glyoxal (40wt % in H_2_O) dissolved in 20 ml of ethanol and the mixture was refluxed for 12 h. At the end of the reaction, the solution was allowed to cool. The orange product obtained was washed with hexane and crystallized from isopropyl alcohol at room temperature (m.p. = 427–430 K, yield 84%). 
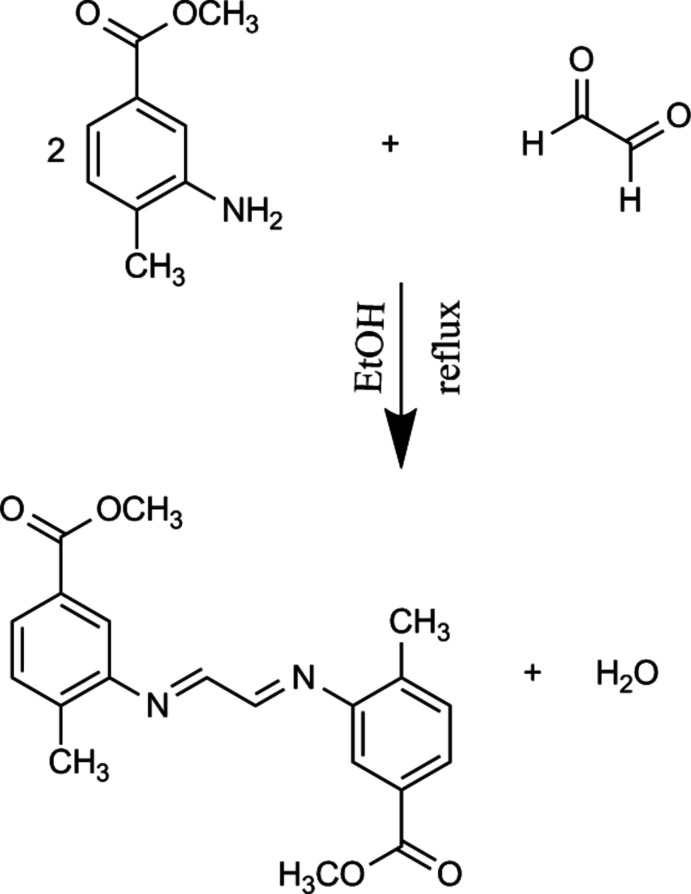


## Refinement

Crystal data, data collection and structure refinement details are summarized in Table 3 ▸. H atoms were positioned geometrically and refined using a riding model: C—H = 0.93–0.97 Å with*U*_iso_(H) = 1.2*U*_eq_(C).

## Supplementary Material

Crystal structure: contains datablock(s) I. DOI: 10.1107/S2056989022002092/zn2013sup1.cif

Structure factors: contains datablock(s) I. DOI: 10.1107/S2056989022002092/zn2013Isup2.hkl

Supporting information file. DOI: 10.1107/S2056989022002092/zn2013Isup3.cml

CCDC reference: 2153984

Additional supporting information:  crystallographic information; 3D view; checkCIF report

## Figures and Tables

**Table 1:** Selected geometric parameters (Å, °)

O2—C2	1.3370 (18)	N1—C7	1.4272 (16)
O2—C1	1.4544 (17)	O1—C2	1.2027 (16)
N1—C10	1.2713 (17)		
			
C2—O2—C1	115.27 (11)	O1—C2—O2	123.25 (13)
			
C10—N1—C7—C8	47.58 (18)		

**Table 2:** Hydrogen-bond geometry (Å, °) *Cg*1 is the centroid of the C3–C8 ring

*D*—H⋯*A*	*D*—H	H⋯*A*	*D*⋯*A*	*D*—H⋯*A*
C10—H10⋯N1^i^	0.93	2.92	3.833 (2)	169
C5—H5⋯O2^ii^	0.93	2.92	3.734 (2)	147
C1—H1*A*⋯O1^iii^	0.96	2.77	3.543 (2)	138
C1—H1*B*⋯O1^iv^	0.96	2.90	3.808 (2)	159
C9—H9*A*⋯*Cg*1^i^	0.96	2.93	3.572 (2)	125

Symmetry codes: (i) 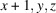 ; (ii) 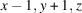 ; (iii) 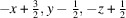 ; (iv) 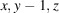 .

**Table 3:** Experimental details

Crystal data	
Chemical formula	C_20_H_20_N_2_O_4_
*M*_r_	352.38
Crystal system, space group	Monoclinic, *P*2_1_/*n*
Temperature (K)	296
*a*, *b*, *c* (Å)	4.6003 (5), 6.2969 (5), 30.726 (4)
β (°)	90.886 (9)
*V* (Å^3^)	889.94 (16)
*Z*	2
Radiation type	Mo *K*α
μ (mm^−1^)	0.09
Crystal size (mm)	0.38 × 0.25 × 0.12

Data collection	
Diffractometer	Stoe IPDS 2
Absorption correction	Integration (*X-RED32*; Stoe & Cie, 2002 ▸)
*T*_min_, *T*_max_	0.971, 0.990
No. of measured, independent and observed [*I* > 2σ(*I*)] reflections	6876, 2002, 1490
*R*_int_	0.036
(sin θ/λ)_max_ (Å^−1^)	0.647

Refinement	
*R*[*F* ^2^ > 2σ(*F* ^2^)], *wR*(*F* ^2^), *S*	0.041, 0.126, 1.06
No. of reflections	2002
No. of parameters	120
H-atom treatment	H-atom parameters constrained
Δρ_max_, Δρ_min_ (e Å^−3^)	0.12, −0.12

Computer programs: *X-AREA* and *X-RED32* (Stoe & Cie, 2002 ▸), *SHELXT2018/3* (Sheldrick, 2015*a* ▸), *SHELXL2018/3* (Sheldrick, 2015*b* ▸), *OLEX2* (Dolomanov *et al.*, 2009 ▸), *Mercury* (Macrae *et al.*, 2020 ▸), *WinGX* (Farrugia, 2012 ▸), *PLATON* (Spek, 2020 ▸) and *publCIF* (Westrip, 2010 ▸).

## supplementary crystallographic information

### Crystal data

**Table:**

C_20_H_20_N_2_O_4_	*F*(000) = 372
*M**_r_* = 352.38	*D*_x_ = 1.315 Mg m^−^^3^
Monoclinic, *P*2_1_/*n*	Mo *K*α radiation, λ = 0.71073 Å
*a* = 4.6003 (5) Å	Cell parameters from 7667 reflections
*b* = 6.2969 (5) Å	θ = 1.3–27.9°
*c* = 30.726 (4) Å	µ = 0.09 mm^−^^1^
β = 90.886 (9)°	*T* = 296 K
*V* = 889.94 (16) Å^3^	Plate, colorless
*Z* = 2	0.38 × 0.25 × 0.12 mm

### Data collection

**Table:**

Stoe IPDS 2 diffractometer	2002 independent reflections
Radiation source: sealed X-ray tube, 12 x 0.4 mm long-fine focus	1490 reflections with *I* > 2σ(*I*)
Plane graphite monochromator	*R*_int_ = 0.036
Detector resolution: 6.67 pixels mm^-1^	θ_max_ = 27.4°, θ_min_ = 1.3°
rotation method scans	*h* = −5→5
Absorption correction: integration (X-RED32; Stoe & Cie, 2002)	*k* = −8→8
*T*_min_ = 0.971, *T*_max_ = 0.990	*l* = −39→39
6876 measured reflections	

### Refinement

**Table:**

Refinement on *F*^2^	Primary atom site location: structure-invariant direct methods
Least-squares matrix: full	Secondary atom site location: difference Fourier map
*R*[*F*^2^ > 2σ(*F*^2^)] = 0.041	Hydrogen site location: inferred from neighbouring sites
*wR*(*F*^2^) = 0.126	H-atom parameters constrained
*S* = 1.06	*w* = 1/[σ^2^(*F*_o_^2^) + (0.0658*P*)^2^ + 0.0582*P*] where *P* = (*F*_o_^2^ + 2*F*_c_^2^)/3
2002 reflections	(Δ/σ)_max_ < 0.001
120 parameters	Δρ_max_ = 0.12 e Å^−^^3^
0 restraints	Δρ_min_ = −0.12 e Å^−^^3^

### Special details

**Table:**

Geometry. All esds (except the esd in the dihedral angle between two l.s. planes) are estimated using the full covariance matrix. The cell esds are taken into account individually in the estimation of esds in distances, angles and torsion angles; correlations between esds in cell parameters are only used when they are defined by crystal symmetry. An approximate (isotropic) treatment of cell esds is used for estimating esds involving l.s. planes.

### Fractional atomic coordinates and isotropic or equivalent isotropic displacement parameters (Å^2^)

**Table:**

	*x*	*y*	*z*	*U*_iso_*/*U*_eq_	
O2	0.9388 (2)	0.49454 (16)	0.32206 (3)	0.0649 (3)	
N1	0.3480 (2)	0.65258 (18)	0.45753 (3)	0.0535 (3)	
O1	0.9663 (3)	0.80450 (19)	0.28689 (4)	0.0830 (4)	
C7	0.4336 (3)	0.7641 (2)	0.41938 (4)	0.0491 (3)	
C3	0.6914 (3)	0.7890 (2)	0.35160 (4)	0.0515 (3)	
C10	0.5415 (3)	0.5584 (2)	0.48034 (4)	0.0517 (3)	
H10	0.7352	0.5633	0.4722	0.062*	
C8	0.6168 (3)	0.6742 (2)	0.38878 (4)	0.0506 (3)	
H8	0.6894	0.5379	0.3931	0.061*	
C6	0.3168 (3)	0.9680 (2)	0.41297 (4)	0.0514 (3)	
C2	0.8798 (3)	0.7010 (2)	0.31694 (4)	0.0562 (3)	
C5	0.3996 (3)	1.0803 (2)	0.37606 (4)	0.0578 (4)	
H5	0.3290	1.2172	0.3717	0.069*	
C4	0.5835 (3)	0.9933 (2)	0.34590 (4)	0.0585 (4)	
H4	0.6356	1.0717	0.3216	0.070*	
C9	0.1143 (3)	1.0662 (3)	0.44519 (5)	0.0653 (4)	
H9A	−0.0500	0.9745	0.4491	0.098*	
H9B	0.0488	1.2014	0.4345	0.098*	
H9C	0.2146	1.0852	0.4725	0.098*	
C1	1.1124 (4)	0.3998 (3)	0.28799 (5)	0.0732 (5)	
H1A	1.0235	0.4286	0.2601	0.110*	
H1B	1.1237	0.2491	0.2924	0.110*	
H1C	1.3046	0.4592	0.2890	0.110*	

### Atomic displacement parameters (Å^2^)

**Table:**

	*U*^11^	*U*^22^	*U*^33^	*U*^12^	*U*^13^	*U*^23^
O2	0.0768 (7)	0.0665 (7)	0.0519 (5)	0.0086 (5)	0.0164 (5)	0.0044 (5)
N1	0.0585 (6)	0.0587 (7)	0.0434 (6)	−0.0063 (5)	0.0068 (5)	0.0059 (5)
O1	0.1034 (9)	0.0811 (8)	0.0656 (7)	0.0040 (6)	0.0373 (6)	0.0176 (6)
C7	0.0512 (7)	0.0560 (7)	0.0402 (6)	−0.0093 (5)	0.0021 (5)	0.0041 (5)
C3	0.0528 (7)	0.0577 (8)	0.0441 (7)	−0.0039 (6)	0.0038 (5)	0.0044 (6)
C10	0.0575 (7)	0.0561 (7)	0.0416 (6)	−0.0072 (6)	0.0067 (5)	0.0021 (6)
C8	0.0535 (7)	0.0528 (7)	0.0455 (6)	−0.0027 (6)	0.0029 (5)	0.0049 (5)
C6	0.0523 (7)	0.0554 (7)	0.0464 (6)	−0.0050 (6)	0.0004 (5)	−0.0012 (6)
C2	0.0578 (8)	0.0647 (8)	0.0463 (7)	−0.0023 (6)	0.0063 (6)	0.0063 (6)
C5	0.0671 (8)	0.0527 (7)	0.0535 (7)	0.0016 (6)	0.0025 (6)	0.0058 (6)
C4	0.0660 (8)	0.0599 (8)	0.0497 (7)	−0.0048 (7)	0.0058 (6)	0.0111 (6)
C9	0.0708 (9)	0.0669 (9)	0.0585 (8)	−0.0017 (7)	0.0097 (7)	−0.0066 (7)
C1	0.0823 (10)	0.0830 (11)	0.0547 (8)	0.0138 (9)	0.0164 (7)	−0.0040 (8)

### Geometric parameters (Å, º)

**Table:**

O2—C2	1.3370 (18)	C8—H8	0.9300
O2—C1	1.4544 (17)	C6—C5	1.3945 (18)
N1—C10	1.2713 (17)	C6—C9	1.5030 (19)
N1—C7	1.4272 (16)	C5—C4	1.378 (2)
O1—C2	1.2027 (16)	C5—H5	0.9300
C7—C8	1.3925 (18)	C4—H4	0.9300
C7—C6	1.4044 (19)	C9—H9A	0.9600
C3—C4	1.389 (2)	C9—H9B	0.9600
C3—C8	1.3991 (17)	C9—H9C	0.9600
C3—C2	1.4903 (19)	C1—H1A	0.9600
C10—C10^i^	1.469 (2)	C1—H1B	0.9600
C10—H10	0.9300	C1—H1C	0.9600
			
C2—O2—C1	115.27 (11)	O2—C2—C3	113.38 (11)
C10—N1—C7	118.87 (11)	C4—C5—C6	121.55 (13)
C8—C7—C6	120.74 (11)	C4—C5—H5	119.2
C8—C7—N1	122.13 (12)	C6—C5—H5	119.2
C6—C7—N1	117.09 (12)	C5—C4—C3	120.36 (12)
C4—C3—C8	119.33 (13)	C5—C4—H4	119.8
C4—C3—C2	117.70 (12)	C3—C4—H4	119.8
C8—C3—C2	122.96 (13)	C6—C9—H9A	109.5
N1—C10—C10^i^	119.86 (16)	C6—C9—H9B	109.5
N1—C10—H10	120.1	H9A—C9—H9B	109.5
C10^i^—C10—H10	120.1	C6—C9—H9C	109.5
C7—C8—C3	119.99 (13)	H9A—C9—H9C	109.5
C7—C8—H8	120.0	H9B—C9—H9C	109.5
C3—C8—H8	120.0	O2—C1—H1A	109.5
C5—C6—C7	117.97 (12)	O2—C1—H1B	109.5
C5—C6—C9	120.47 (13)	H1A—C1—H1B	109.5
C7—C6—C9	121.54 (12)	O2—C1—H1C	109.5
O1—C2—O2	123.25 (13)	H1A—C1—H1C	109.5
O1—C2—C3	123.36 (14)	H1B—C1—H1C	109.5
			
C10—N1—C7—C8	47.58 (18)	C1—O2—C2—O1	−1.2 (2)
C10—N1—C7—C6	−134.55 (13)	C1—O2—C2—C3	177.61 (12)
C7—N1—C10—C10^i^	−179.71 (14)	C4—C3—C2—O1	7.9 (2)
C6—C7—C8—C3	1.39 (19)	C8—C3—C2—O1	−173.12 (14)
N1—C7—C8—C3	179.18 (11)	C4—C3—C2—O2	−170.92 (12)
C4—C3—C8—C7	0.6 (2)	C8—C3—C2—O2	8.1 (2)
C2—C3—C8—C7	−178.35 (12)	C7—C6—C5—C4	1.8 (2)
C8—C7—C6—C5	−2.60 (19)	C9—C6—C5—C4	−179.56 (13)
N1—C7—C6—C5	179.50 (12)	C6—C5—C4—C3	0.1 (2)
C8—C7—C6—C9	178.82 (13)	C8—C3—C4—C5	−1.4 (2)
N1—C7—C6—C9	0.93 (18)	C2—C3—C4—C5	177.63 (13)

Symmetry code: (i) −*x*+1, −*y*+1, −*z*+1.

### Hydrogen-bond geometry (Å, º)

*Cg*1 is the centroid of the C3–C8 ring

**Table:**

*D*—H···*A*	*D*—H	H···*A*	*D*···*A*	*D*—H···*A*
C10—H10···N1^ii^	0.93	2.92	3.833 (2)	169
C5—H5···O2^iii^	0.93	2.92	3.734 (2)	147
C1—H1*A*···O1^iv^	0.96	2.77	3.543 (2)	138
C1—H1*B*···O1^v^	0.96	2.90	3.808 (2)	159
C9—H9*A*···*Cg*1^ii^	0.96	2.93	3.572 (2)	125

Symmetry codes: (ii) *x*+1, *y*, *z*; (iii) *x*−1, *y*+1, *z*; (iv) −*x*+3/2, *y*−1/2, −*z*+1/2; (v) *x*, *y*−1, *z*.
